# Pembrolizumab‐induced secondary sclerosing cholangitis in a non‐small cell lung cancer patient

**DOI:** 10.1002/rcr2.560

**Published:** 2020-04-10

**Authors:** Sachiko Matsumoto, Keisuke Watanabe, Nobuaki Kobayashi, Kuniyasu Irie, Shoji Yamanaka, Takeshi Kaneko

**Affiliations:** ^1^ Department of Pulmonology Yokohama City University Graduate School of Medicine Yokohama Japan; ^2^ Division of Gastroenterology Yokohama City University Graduate School of Medicine Yokohama Japan; ^3^ Department of Pathology Yokohama City University Graduate School of Medicine Yokohama Japan

**Keywords:** ICI, immune‐related adverse events, non‐small cell lung cancer, pembrolizumab, sclerosing cholangitis

## Abstract

A 50‐year‐old woman with stage IV lung adenocarcinoma received seven cycles of pembrolizumab as third‐line chemotherapy. Following the failure of pembrolizumab, she commenced fourth‐line chemotherapy of docetaxel and ramucirumab. The patient complained of epigastric pain and a computed tomography (CT) scan revealed oedema‐like thickening of the gallbladder wall, dilation of the bile ducts from the common to the intrahepatic bile ducts, and thickening of the common bile duct wall without any visible obstructions. Accumulation of fluorodeoxyglucose (FDG) in the gallbladder wall and bile duct was also detected with positron emission tomography (PET)‐CT. A biopsy of the extrahepatic bile duct showed non‐specific inflammation. Antibiotic treatment was not effective and pathogens were not detected. The patient was diagnosed with secondary sclerosing cholangitis (SSC) by pembrolizumab. She received 80 mg/day of prednisolone (PSL); however, SSC recurred with tapering of PSL. SSC then improved with steroid pulse therapy and subsequently 50 mg/day azathioprine and 80 mg/day PSL.

## Graphical abstract

Pembrolizumab, a humanized anti‐programmed cell death‐1 (PD‐1) monoclonal antibody, demonstrates efficacy in treating non‐small cell lung cancer (NSCLC). However, it can cause immune‐related adverse events (irAEs), including inflammatory manifestations. Cholangitis is a rare irAE. Herein, we report on an NSCLC patient who developed pembrolizumab‐induced cholangitis and review the relevant literature.

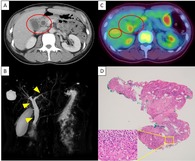

## Introduction

Immune checkpoint inhibitors (ICIs), including pembrolizumab, efficiently treat various malignancies. Pembrolizumab, a humanized anti‐programmed cell death‐1 (PD‐1) monoclonal antibody, demonstrates efficacy in treating non‐small cell lung cancer (NSCLC) 1. However, these inhibitors can cause immune‐related adverse events (irAEs), including inflammatory manifestations. Cholangitis is a rare irAE. Herein, we report on an NSCLC patient who developed pembrolizumab‐induced cholangitis and review the relevant literature.

## Case Report

**Figure 1:**
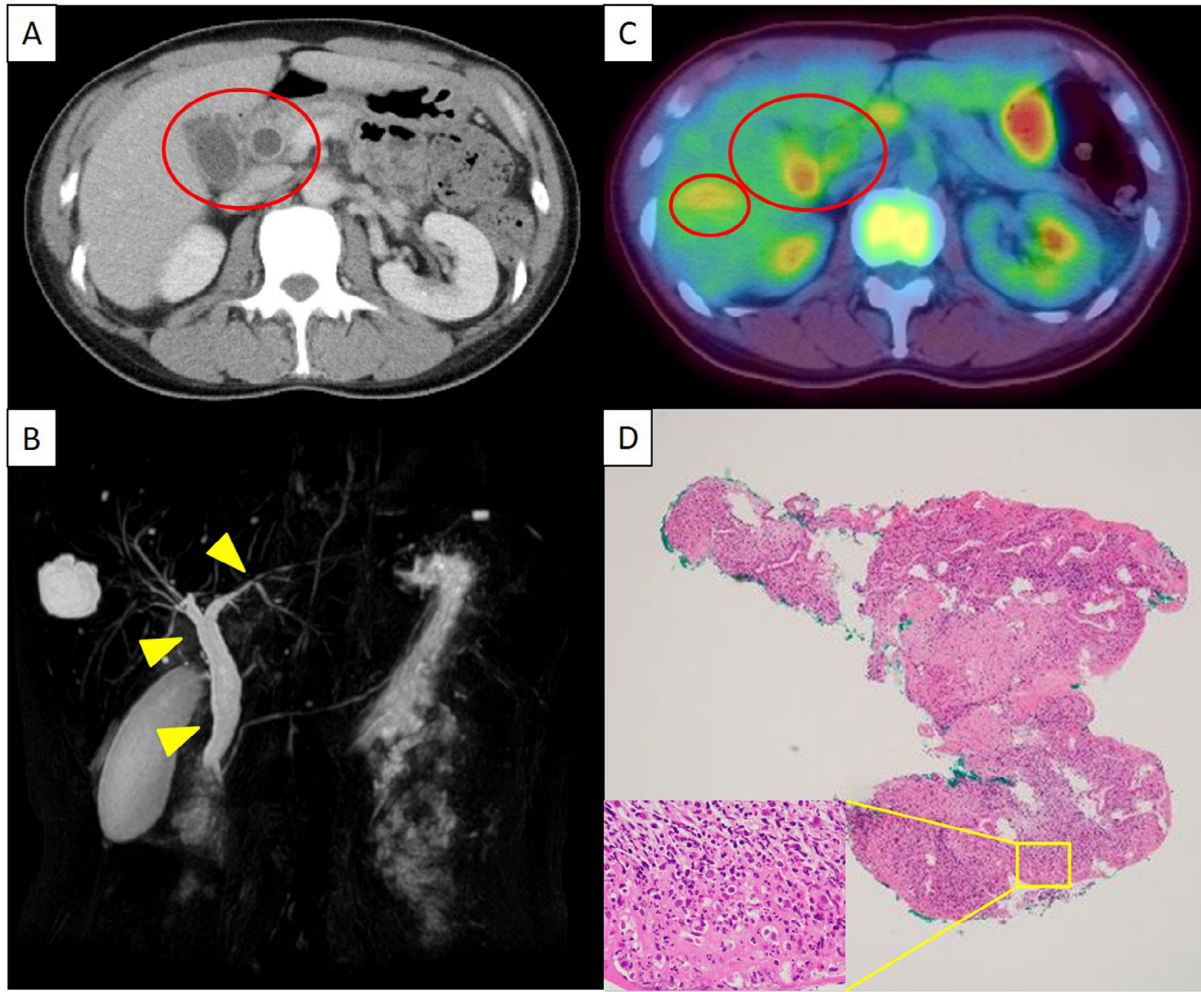
Diagnostic images of a 50‐year‐old Japanese woman diagnosed with lung adenocarcinoma. (A) Enhanced computed tomography (CT) scan revealed oedema‐like thickening of the gallbladder wall, dilation of the bile duct from the common bile duct to the intrahepatic bile ducts, and thickening of the common bile duct wall without any visible obstructions. (B) Magnetic resonance cholangiopancreatography showed irregularly narrowed intrahepatic bile ducts and dilation of peripheral bile ducts. (C) Accumulation of fluorodeoxyglucose (FDG) in the wall of gallbladder and bile duct was also detected with positron emission tomography (PET)‐CT. (D) Biopsy of the extrahepatic bile duct showed numerous neutrophils and lymphoid infiltrates as non‐specific inflammation.

A 50‐year‐old Japanese woman with a past smoking history was diagnosed with lung adenocarcinoma (cT1bN3M1b stage IV) in July 2016. Her lung cancer had a K‐Ras mutation and had metastasized to the pleura, lymph nodes, liver, adrenal glands, and pancreas. She received four cycles of combination chemotherapy with carboplatin (CBDCA), pemetrexed (PEM), and bevacizumab (BEV) in September 2016 followed by 14 cycles of PEM and BEV as maintenance chemotherapy from December 2016 to October 2017, and S‐1 as second‐line chemotherapy from December 2017 to June 2018. Following these treatments, an 18F‐fluorodeoxyglucose (FDG) positron emission tomography (PET)‐computed tomography (CT) scan showed increased accumulation in the left and right neck and supraclavicular and axillary lymph nodes as well as sclerotic changes in the left iliac bone, suggesting bone metastasis. PD‐L1 tumor proportion score (TPS) was 70% positive in a specimen from the left cervical lymph node; therefore, the patient received seven cycles of pembrolizumab as third‐line chemotherapy. Afterward, FDG accumulation was found in the left submandibular region, near the oesophagus, left hilar region, right inguinal region, and mesenteric lymph node, and was judged to be progressive disease (PD). The patient started fourth‐line chemotherapy of docetaxel (DOC) and ramucirumab (RAM) in December 2018. After the second cycle, she complained of epigastric pain and laboratory findings revealed elevated levels of aspartate aminotransferase (AST; 73 IU/L), alanine aminotransferase (ALT; 50 IU/L), alkaline phosphatase (ALP; 1289 IU/L), and γ‐glutamyl transferase (GGT; 370 IU/L). Total bilirubin (0.3 mg/dL), serum immunological markers including antinuclear antibody (ANA), antimitochondrial antibody (AMA), immunoglobulin G4 (IgG4), and myeloperoxidase antineutrophil cytoplasmic antibody were within the normal range. Anti‐SS‐B antibodies were present; however, she had no symptoms of Sjogren's syndrome. A contrast‐enhanced CT scan revealed oedema‐like thickening of the gallbladder wall, dilation of the bile ducts from the common bile duct to intrahepatic bile ducts, and thickening of the common bile duct wall without any visible obstructions, including cholelithiasis or tumours (Fig. 1A). Magnetic resonance cholangiopancreatography showed irregularly narrowed intrahepatic bile ducts and dilation of peripheral bile ducts (Fig. 1B). Accumulation of FDG in the wall of the gallbladder and bile duct was also detected with PET‐CT. A biopsy of the extrahepatic bile duct showed numerous neutrophils and lymphoid infiltrates as non‐specific inflammation (Fig. 1D). Antibiotic treatment with meropenem was not effective and pathogens were not detected from blood and bile cultures. According to these findings and the clinical course of lung adenocarcinoma, the patient was diagnosed with secondary sclerosing cholangitis (SSC) induced by pembrolizumab and began treatment with 80 mg/day prednisolone (PSL). During the PSL tapering, biliary enzymes increased again. Steroid pulse therapy (methyl PSL 1 g for three days) was performed and subsequently, treatment with 50 mg/day azathioprine and 80 mg/day PSL was initiated. The biliary enzymes gradually decreased. Serial examination showed improvement in the wall thickening of the bile duct and gallbladder. FDG accumulation in the wall of the gallbladder and bile duct also disappeared.

## Discussion

ICIs have led to remarkable advances in the treatment of cancers, including NSCLC. However, they can cause irAEs 2. Cholangitis is a rare irAE and four cases of pembrolizumab‐induced SSC in NSCLC patients have been reported 3, 4, 5.

Anti‐PD‐1 antibody‐related cholangitis was mainly reported in NSCLC patients receiving nivolumab (Table 1) 3, 4, 5, 6, 7, 8, 9, 10, 11, 12, 13, 14. Nivolumab‐related cholangitis was characterized by (1) extrahepatic bile duct dilation without obstruction; (2) diffuse hypertrophy of the extrahepatic bile duct wall; (3) increase in the biliary enzymes ALP and GGT relative to the hepatic enzymes AST and ALT; (4) absence of the serum immunological markers ANA, AMA, anti‐smooth muscle antibody, and IgG4; (5) CD8^+^ T cell infiltration in the portal area by liver biopsy; and (6) a moderate‐to‐poor response to steroid therapy 6. Some cases responded well to steroid therapy or improved with discontinuation of anti‐PD‐1 antibodies (Table 1). Further study is needed to fully understand cholangitis induced by anti‐PD‐1 antibodies.

**Table 1:** Summary of reported cases of PD‐L1 inhibitor‐induced cholangitis in non‐small cell lung cancer.

Age	Sex	Drugs	Histology	Symptom	Initiation to occurrence	Elevation of ALP/GGT	Elevation of bilirubin	Treatment	Response	Ref
52	M	Nivo	Ad	Abdominal pain	8 cycles	+	−	PSL 0.5 mg/kg	Yes	3
N/A	M	Durva	Sq	Fever, abdominal pain	4 cycles	N/A	N/A	PSL 120 mg	Yes	3
61	M	Pem	Ad	None	17 cycles	N/A	N/A	PSL 1 mg/kg	Yes	3
68	M	Pem	Ad	Abdominal pain, vomiting	5.5 months	+	−	PSL 50 mg	Yes	4
67	M	Pem	NSCLC	Fever, malaise	1 month	+	−	PSL 40 mg	Yes	4
63	M	Pem	NSCLC	None	7 cycles	+	−	PSL 1 mg/kg	Yes	5
55	M	Nivo	NSCLC	Abdominal pain	11 cycles	+	−	mPSL 2 mg/kg, MMF 2 g	Yes	5
81	F	Nivo	NSCLC	Backache	25 cycles	+	−	mPSL 2 mg/kg, MMF 2 g	Yes	5
82	F	Nivo	NSCLC	None	2 cycles	+	−	mPSL 1.6 mg/kg	Yes	5
64	M	Nivo	Ad	Fever, abdominal discomfort	9 cycles	+	−	PSL 30 mg	No	6
73	F	Nivo	Sq	Fever, vomiting, abdominal discomfort, diarrhoea	6 cycles	+	+	PSL 30 mg	Yes	6
83	F	Nivo	Sq	Fever, general fatigue	12 cycles +2 months	+	−	Discontinuation	Yes	6
63	M	Nivo	Ad	Fever, abdominal discomfort	5 cycles	+	−	PSL 2 mg/kg	Yes	7
69	M	Nivo	Ad	Rash	3 cycles	−	+	PSL 50 mg, mPSL 500 mg	Yes	8
57	F	Nivo	Sq	Abdominal discomfort	7 cycles + 7 months	+	−	Discontinuation	Yes	9
79	M	Nivo	NSCLC	Itching, jaundice	62 days	+	+	mPSL 1 mg/kg	Yes	10
56	F	Nivo	Ad	Myalgia, diffuse skin thickening	16 cycles	+	−	Corticosteroids	N/A	11
69	M	Avel	Ad	Right upper abdominal discomfort	21 cycles	+	−	mPSL 1 mg/kg	Yes	12
71	M	Nivo	NSCLC	None	11 months	+	−	mPSL 0.5 mg/kg	Yes	13
67	M	Nivo	Mixed Ad and Sq	Right upper abdominal pain	8 cycles	+	N/A	mPSL, PSL 50 mg, MMF, Tacrolimus	No	14
50	F	Pem	Ad	Epigastric pain	7 cycles	+	−	PSL 80 mg, azathioprine 50 mg	Yes	Present case

Ad, adenocarcinoma; ALP, alkaline phosphatase; Avel, avelumab; Durva, durvalumab; GGT, γ‐glutamyl transferase; MMF, mycophenolate mofetil; mPSL, methyl‐PSL; N/A, not available; Nivo, nivorlumab; NSCLC, non‐small cell lung cancer; PD‐L1, programmed death‐ligand 1; Pem, pembrolizumab; PSL, prednisolone; Ref, reference; Sq, squamous cell lung cancer.

In addition to SSC induced by pembrolizumab, SSC caused from paraneoplastic syndrome should be considered during treatment with ICIs. Milkiewicz et al. reported six patients with neoplastic diseases who acquired autoimmune liver diseases and suggested the possibility of paraneoplastic syndrome 15. Testing for antibodies that are specific to paraneoplastic syndrome, such as anti‐Hu antibodies, could have been useful in this case to elucidate the reason for cholangitis.

Our patient was positive for anti‐SS‐B antibodies before receiving pembrolizumab. Anti‐PD‐1 antibody is efficient and safe for patients with advanced NSCLC 16. Anti‐PD‐1 antibody has also been tested to determine its influence on ANA 16. Although the incidence of irAEs was not significantly different between ANA‐positive and ‐negative cases, the incidence of irAEs tended to increase as the ANA titre increased. Furthermore, the frequency of irAEs including infusion reaction, pneumonitis, and arthralgia after administration of ICIs was high in NSCLC patients positive for ANA and rheumatoid factor (RF), although no patients developed autoimmune disease after ICIs were administered 17. Owing to these reports, our patient's irAE may be related to her positive anti‐SS‐B antibody status.

A PET‐CT showed FDG accumulation in the wall of the gallbladder and bile duct. There are no reports of PET‐CT scans identifying anti‐PD‐L1 antibody‐induced cholangitis; however, PET‐CT is useful for detecting some irAEs and early detection of cholangitis might be possible with PET‐CT 18.

ICIs have clinical benefits; however, they have a risk of very rare adverse events like SSC that may go unreported. Further studies are necessary to establish the risk of SSC in patients receiving ICIs and the optimal management of ICI‐induced SSC.

### Disclosure Statement

Appropriate written informed consent was obtained for publication of this case report and accompanying images.
